# The dyslexia susceptibility *KIAA0319* gene shows a specific expression pattern during zebrafish development supporting a role beyond neuronal migration

**DOI:** 10.1002/cne.24696

**Published:** 2019-04-16

**Authors:** Monika Gostic, Angela Martinelli, Carl Tucker, Zhengyi Yang, Federico Gasparoli, Jade‐Yi Ewart, Kishan Dholakia, Keith T. Sillar, Javier A. Tello, Silvia Paracchini

**Affiliations:** ^1^ School of Medicine University of St Andrews St Andrews UK; ^2^ Biomedical Sciences Research Complex University of St Andrews St Andrews UK; ^3^ College of Medicine and Veterinary Medicine The University of Edinburgh Edinburgh UK; ^4^ SUPA, School of Physics and Astronomy University of St Andrews St Andrews UK; ^5^ School of Psychology and Neuroscience University of St Andrews St Andrews UK

**Keywords:** dyslexia, gene expression, KIAA0319, neurodevelopment, notochord, RRID:SCR_001783, RRID:SCR_003070, RRID:SCR_012481, zebrafish

## Abstract

Dyslexia is a common neurodevelopmental disorder caused by a significant genetic component. The *KIAA0319* gene is one of the most robust dyslexia susceptibility factors but its function remains poorly understood. Initial RNA‐interference studies in rats suggested a role in neuronal migration whereas subsequent work with double knock‐out mouse models for both *Kiaa0319* and its paralogue *Kiaa0319‐like* reported effects in the auditory system but not in neuronal migration. To further understand the role of *KIAA0319* during neurodevelopment, we carried out an expression study of its zebrafish orthologue at different embryonic stages. We used different approaches including RNAscope in situ hybridization combined with light‐sheet microscopy. The results show particularly high expression during the first few hours of development. Later, expression becomes localized in well‐defined structures. In addition to high expression in the brain, we report for the first time expression in the eyes and the notochord. Surprisingly, *kiaa0319‐like,* which generally shows a similar expression pattern to *kiaa0319*, was not expressed in the notochord suggesting a distinct role for *kiaa0319* in this structure. This observation was supported by the identification of notochord enhancers enriched upstream of the *KIAA0319* transcription start site, in both zebrafish and humans. This study supports a developmental role for *KIAA0319* in the brain as well as in other developing structures, particularly in the notochord which, is key for establishing body patterning in vertebrates.

## 1 INTRODUCTION

Developmental dyslexia is a specific impairment in learning to read in the absence of any other obvious impairing factors. It affects at least 5% of school‐aged children and its heritability is estimated to be above 60% (Shaywitz & Shaywitz, 2005). Studying the genetic contribution to dyslexia may help to dissect the underlying neuropsychological mechanisms, which remain hotly debated (Goswami, 2014). While a phonological deficit is the most commonly accepted cause for dyslexia, sensory dysfunction in the visual and auditory systems have also been observed in a number of studies (Goswami, 2014; Ramus & Ahissar, 2012; Shaywitz & Shaywitz, 2005).

The *DYX1C1, DCDC2, ROBO1,* and *KIAA0319* genes are known as the classical dyslexia susceptibility genes and they are supported by a number of independent replication studies (Carrion‐Castillo, Franke, & Fisher, 2013; Newbury, Monaco, & Paracchini, 2014). A role in cortical development, and specifically in neuronal migration, has been proposed for these genes (Paracchini, Scerri, & Monaco, 2007), in line with earlier postmortem observations that reported subtle cortical defects in individuals with dyslexia (Galaburda, LoTurco, Ramus, Fitch, & Rosen, 2006; Humphreys, Kaufmann, & Galaburda, 1990). In particular, *KIAA0319* variants have been found to be associated with dyslexia and reading abilities in multiple clinical and epidemiological cohorts (Newbury et al., 2014; Paracchini, 2011). A specific dyslexia‐associated variant was shown to affect *KIAA0319* transcription regulation and gene expression levels, providing a mechanism to link genetic variation with the disorder (Dennis et al., 2009; S Paracchini et al., 2006). Its paralogous gene, *KIAA0319‐LIKE* or *KIAA0319L*, has also been reported to be associated with dyslexia but with weaker evidence (Couto et al., 2008). Both *KIAA0319* and *KIAA0319L* are transmembrane proteins (Velayos‐Baeza, Toma, da Roza, Paracchini, & Monaco, 2007), but their exact cellular functions remain unclear.

A new role in cilia biology is emerging for dyslexia candidate genes (Brandler & Paracchini, 2014; Paracchini, Diaz, & Stein, 2016). A transcriptome study showed differential regulation for *KIAA0319, DCDC2,* and *DYX1C1* in ciliated tissues (Ivliev, t Hoen, van Roon‐Mom, Peters, & Sergeeva, 2012). Functional studies for Dyx1c1 and Dcdc2 showed a role in ciliogenesis in different biological models, including zebrafish, (Chandrasekar, Vesterlund, Hultenby, Tapia‐Paez, & Kere, 2013; Massinen et al., 2011; Tarkar et al., 2013) and some patients with ciliopathies have been found to harbor mutations in both genes (Schueler et al., 2015; Tarkar et al., 2013). While there is no direct evidence supporting a role for KIAA0319 in cilia, the presence of five PKD domains in KIAA0319 lends support to this notion (Velayos‐Baeza, Toma, Paracchini, & Monaco, 2008). Mutations in PKD genes, which play key roles in cilia, lead to ciliopathies and laterality defects (Hildebrandt, Benzing, & Katsanis, 2011). *KIAA0319* has been shown to be a target of T‐Brain‐1 (TBR1), a transcription factor implicated in autism which regulates different brain developmental processes, such as neuronal migration, axon guidance (Chuang, Huang, & Hsueh, 2015) and the determination of left–right asymmetries in bilaterians (Kitaguchi, Mizugishi, Hatayama, Aruga, & Mikoshiba, 2002). *KIAA0319* has been shown to be involved in axon growth and regeneration supporting a role in the adult peripheral nervous system (Franquinho et al., 2017).

Both *KIAA0319* and *KIAA0319L* have been implicated in neuronal migration following knockdown experiments that specifically targeted neurons at the early stages of brain development using in utero shRNA in rats (Adler et al., 2013; Paracchini et al., 2006; Peschansky et al., 2010; Platt et al., 2013; Szalkowski et al., 2012). However, knockout (KO) mouse models do not display any cortical abnormalities that could be explained by defective neuronal migration (Guidi et al., 2017; Martinez‐Garay et al., 2017). Instead, the KO mice presented auditory system defects (Guidi et al., 2017) in line with observations reported in adult rats that underwent KIAA0319 knock‐down in utero (Centanni et al., 2014; Centanni et al., 2014; Szalkowski et al., 2012). Therefore, while a role for *KIAA0319* in neurodevelopment is supported by different lines of evidence, its exact function remains largely unclear.

Here, we report a gene expression study for the *kiaa0319* gene in zebrafish to further understand the role of this gene during vertebrate development. We observed a spatiotemporal expression pattern beyond the brain including in the eyes and the notochord. Surprisingly, *kiaa0319‐like,* which presents a widespread expression in other species, was not expressed in the notochord, suggesting role specific to *kiaa0319*. These data support a role for *KIAA0319* both in the brain and in other structures and suggest for the first time a function in the notochord.

## 2 MATERIALS AND METHODS

### 2.1 Fish care

All the experimental procedures were approved by the Animal Welfare Ethics Committee at the University of St Andrews in compliance with the Home Office regulations. All researchers who conducted work with animals held a Personal License issued by the Home Office.

Wild type zebrafish (*Danio rerio*) (WIK and AB/TU) and the double transgenic Tg(gfap:GFP);Tg(Oligo2:dsRed) were raised at The Queen's Medical Research Institute at the University of Edinburgh according to standard procedures in a home office approved facility. Developmental stages, maintained at 28.5°C, were identified as previously described (Kimmel, Ballard, Kimmel, Ullmann, & Schilling, 1995). Animals were handled in accordance with the guidelines from European Directive 2010/63/EU and euthanised in accordance with Schedule 1 procedures of the Home Office Animals (Scientific Procedures) Act 1986. Zebrafish embryos were obtained using the Mass Embryo production system (MEPs) of the wild type line Wik.

### 2.2 PCR and qPCR

Total RNA from developmental stages between 16 and 32 cells, up to 120 hour post‐fertilization (hpf) was extracted using the RNeasy Mini kit according to the manufacturer's instructions (QIAGEN) using at least 50 embryos at each stage. The heart, liver and brain were dissected from five adult fish, flash‐frozen on dry ice and stored at −80°C until the RNA was extracted. Eyes were dissected (*N* = 40 eyes total) at 48 hpf stage and flash frozen on dry ice.

The PrimeScript RT reagent kit (Takara) was used to transcribe the RNA into the cDNA following the manufacturer's protocol. The presence of *kiaa0319* transcripts was verified by electrophoresis following PCR amplification. Gene expression was assessed by quantitative PCR (qPCR) conducted with the Luna Universal RT‐qPCR Kit (NEB) and using a Viia7 instrument (Life Technologies, Paisley, UK). For protocol details see Supplementary materials. Primer sequences and accession numbers are shown in Table S1. Differences in gene expression were evaluated with the Wilcoxon‐Mann–Whitney test implemented in R (RStudioTeam, 2015).

### 2.3 Whole‐mount in situ hybridization

Whole‐mount in situ hybridization (WISH) was carried out following a previously described protocol (Thisse & Thisse, 2008). Briefly, a DIG‐labeled riboprobe targeting *kiaa0319* was transcribed using a T3 Polymerase with a DIG RNA Labeling Mix (Roche) according to the manufacturer's instructions and, as a template, a 1,066 bp PCR fragment amplified from cDNA (Table S1). Zebrafish embryos were collected and processed at 3 somite, 14 somite, 30 hpf, and 48 hpf. Embryos were imaged with a Leica MZ16F or MZFLIII bright field microscopes following treatment with an anti‐DIG antibody (Roche, diluted 1:5000 in blocking buffer) and a staining solution (NBT and BCIP, Roche).

### 2.4 RNAscope

RNAscope in situ hybridization (ISH; Advanced Cell Diagnostics; RRID:SCR_012481) was modified from a previously described protocol (Gross‐Thebing, Paksa, & Raz, 2014). Samples were fixed in 4% PFA at room temperature for a length of time dependent on the developmental stage (Table S2). Samples were hybridized with RNAscope target probes (*kiaa0319l*, nt 545‐1425 of ENSDART00000051723.5, Channel 1; *myoD1*, nt 2‐1083 of ENSDART00000027661.7, Channel 2; *kiaa0319*, nt 239‐1147 of ENSDART00000160645, Channel 3) overnight at 40°C. Images were taken with a Leica TCS SP8 confocal microscope under ×20 magnification and processed in Leica Application Software X (LAS X), unless otherwise specified. Light sheet microscopy (LSM) was conducted with a bespoke microscope built in‐house (Supplementary Materials). Images were manipulated with ImageJ (RRID:SCR_003070).

### 2.5 Sequence analysis

The zebrafish orthologues of the human KIAA0319 and KIAA0319L genes were identified in the zebrafish genome using the UCSC genome browser (Kent et al., 2002). The 10 kb regions upstream of the zebrafish and human *KIAA0319* (danRer10 chr16:36946809–36952809; hg38 range = chr6:24645946–24652021) and *KIAA0319L* (danRer10 range = chr19:4401525–44013525; hg38 range = chr1:35557338–35563413) transcription start sites (TSS) were scanned for the presence of *FOXA2* (a key regulator for genes expressed in the notochord [Tamplin, Cox, & Rossant, 2011]) consensus sequences (5′‐T[G/A]TTT[A/G][C/T]T‐3′) with the FIMO software from the MEME suite (RRID:SCR_001783) (Grant, Bailey, & Noble, 2011).

## 3 RESULTS

### 3.1 Exploratory analysis

**Figure 1:**
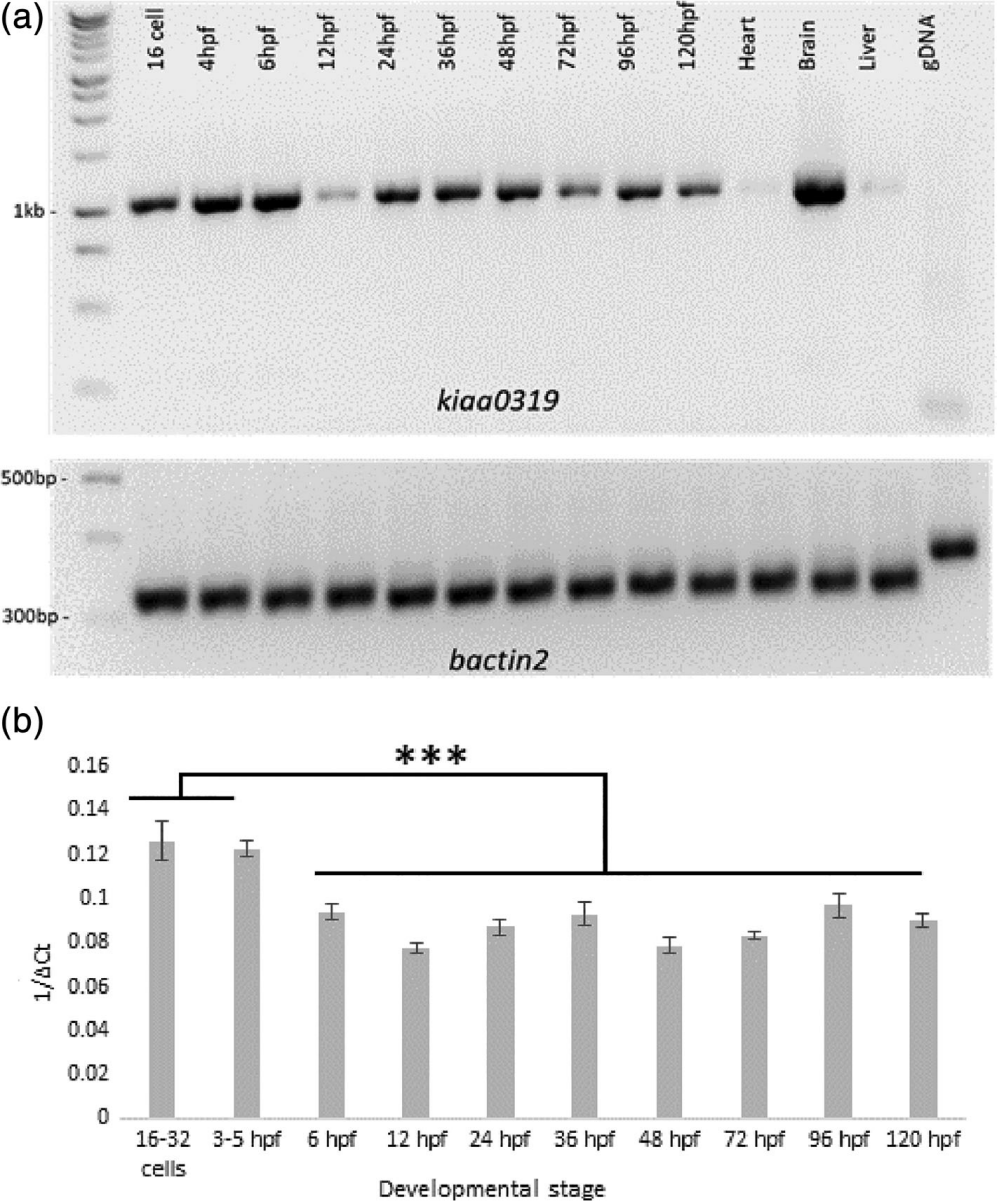
*kiaa0319* is expressed during different stages of development. (a) The top panel shows RT‐PCR amplicons generated with primers targeting different *kiaa0319* exons using cDNA prepared at different zebrafish developmental stages (hours post‐fertilization, hpf) and in selected adult tissues. The amplicons have the expected size of 1,024 bp. The lower panel shows fragments (322 bp) for *bactin2* used as a control for cDNA quality at the corresponding stages and tissues. Genomic DNA (gDNA) in the last lane demonstrates the specificity of the assay with no band for the *kiaa0319* reaction and a fragment of 407 bp for the *bactin2* as expected. *kiaa0319* is expressed throughout the different development stages and in the adult brain but with only weak signal in the hearth and liver. The adult data are consistent with the expression profiles observed in human adult tissue (Figure S1). The top and lower panel are images from two separate gels where samples were loaded in the same order. (b) Quantification of the expression of *kiaa0319* by qPCR measured during the first 5 days of development. Expression is measured against the *eef1a1l2* gene, used as reference. Mean values are derived from biological triplicates and error bars indicate standard deviations. The first two time points showed significantly higher expression compared to later stages (*p* < .0001)

As a first step of our analysis, we verified the expression of *kiaa0319* during early zebrafish development using RT‐PCR (Figure 1a). The expression of *kiaa0319* was observed across all the developmental stages that were analyzed. In the adult, expression was much higher in the brain compared to heart and liver, where it is barely detectable. The high expression in the brain is consistent with the expression profile observed for *KIAA0319* in humans in contrast to *KIAA0319L,* which is widespread across human tissues (Figure S1). Quantification of expression by qPCR confirmed *kiaa0319* expression at different developmental stages and showed that the highest level was detected in the earliest stages of development (Figure 1b), whereas the lower expression was observed at 12 hpf. Expression at the first development stages (up to 5 hpf) was significantly higher compared to later time points (*p* < .0001).

To localize *kiaa0319* expression patterns we conducted WISH (Figure S2). Consistent with the qPCR data, we observed high *kiaa0319* expression during the early stages of embryonic development (Figure S2). As development progresses, this widespread expression becomes restricted to specific structures. At the 14 somite stage (16 hpf), *kiaa0319* expression can be visualized in the developing brain and the body midline (Figures S2a3 and S2a4). At 30 hpf, expression is detected in the eye, the otic vesicle, and in the midbrain‐hindbrain boundary (Figure S2b). The midline expression appears localized to the notochord rather than the spinal cord. At 48 hpf expression becomes weaker in the eyes and otic vesicles and is more pronounced in the telencephalon (Figure S2c). WISH confirmed expression of *kiaa0319* both in the brain, as expected, as well as in other tissues where a role for KIAA0319 has not been described before.

### 3.2 RNAscope analysis

**Figure 2:**
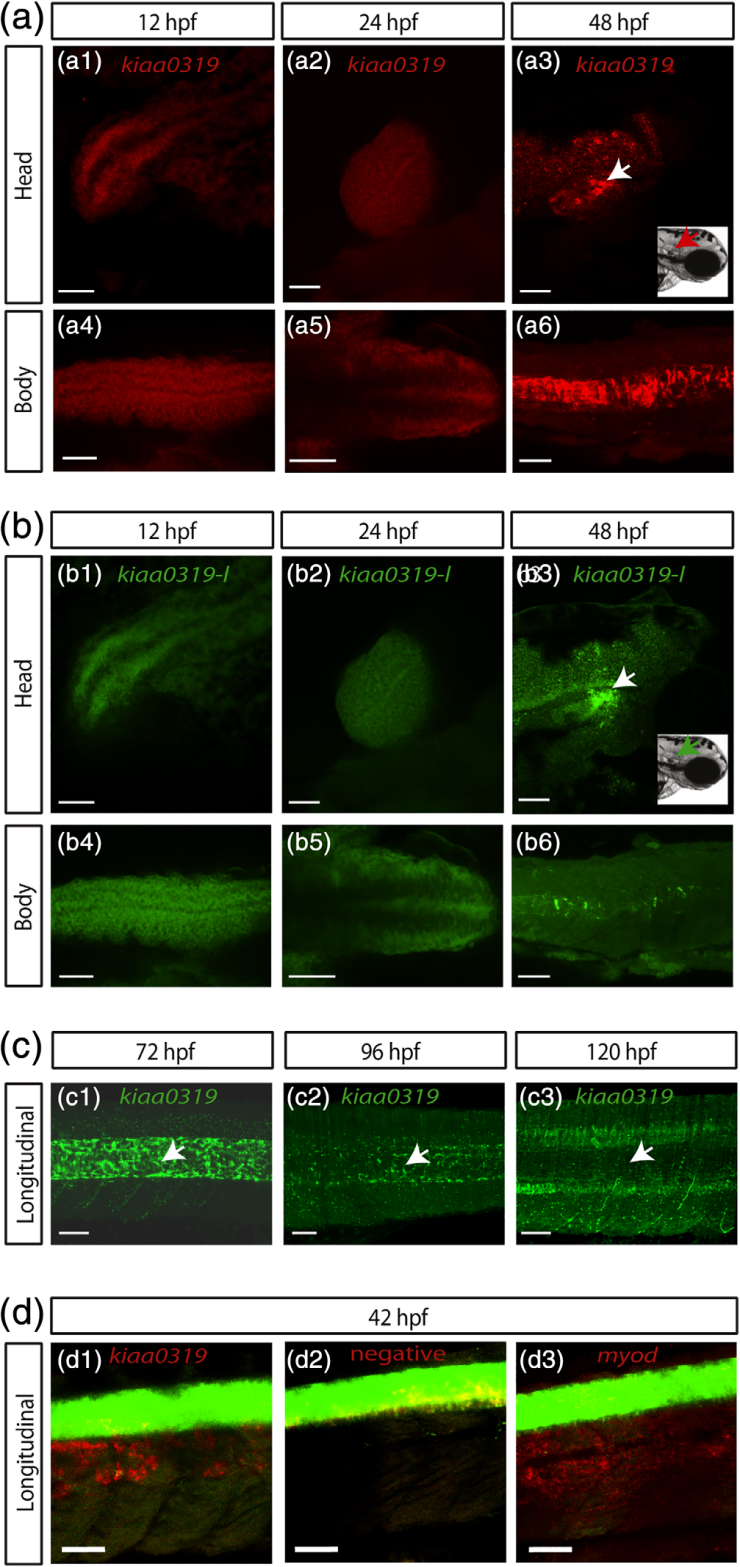
*kiaa0319* is specifically expressed in the notochord. The expression of *kiaa0319* (a) and *kiaa0319l* (b) was examined at three embryonic stages (12, 24, and 48 hpf; WT zebrafish) using the RNAScope fluorescent multiplex assay with results shown for the head (a1, a2, a3, b1, b2, and b3) and the body (a4, a5, a6, b4, b5, and b6). *kiaa0319* (red) is expressed throughout the three stages. High expression is detected in the developing brain and body midline. At 48 hpf, *kiaa0319* is still highly expressed in the brain and strong signal is detected in the otic vesicles (a3; white arrows corresponding to the red arrow in the reference image) and in the notochord (a6). *kiaa0319l* shows a similar pattern of expression, including a strong signal in the otic vesicles (b3), but the expression in the notochord at 48hpf is very weak (b6). Black area in the brain at 48 hpf correspond to the pigmentation of the embryo (a3 and b3). (c) Longitudinal view of the body with 3D reconstructions from light‐sheet microscopy images at three developmental stages 72 (c1), 96 (c2), and 120(c3) hpf. The samples from a WT zebrafish are labeled with the *kiaa0319* probe. *kiaa0319* expression is localized to the notochord (white arrow). The signal diminishes as development progresses and the notochord regresses. See a 3D animation at 72hpf that allows assessing the signal from different orientations (Video SV1). See Figure S3 for the positive and negative controls acquired with light sheet microscopy (d) *kiaa0319* expression in the notochord was confirmed with the Tg(gfap:GFP);Tg(Oligo2:dsRed) transgenic line, which express GFP (green) in the spinal cord (d1). No signal was detected for the negative control (d2) *myoD1* (myogenic differentiation 1, a universal target for myogenic cells [Weinberg et al., 1996]) was used as positive control and demonstrates the specificity of the assay images were collected at 42 hpf by confocal microscopy. The scale bar indicates 50 μm in all panels

To verify this expression pattern and to achieve higher resolution, and specificity we used the highly sensitive RNAscope fluorescent multiplex assay (Figure 2). In particular, we focused on tissues other than brain. These included the body midline, the otic vesicles and the eyes following the observations made by WISH. For comparison, we included in the analysis the *kiaa0319‐like* gene. Consistent with the qPCR and the WISH results, at 24 hpf *kiaa0319* expression is widespread but stronger in the brain and body midline (Figure 2a). Expression in specific structures, such as the otic vesicles was visible at 48 hpf. The *kiaa0319‐like* presented a similar pattern of expression but, surprisingly, a much weaker signal was observed in the notochord (Figure 2b). Therefore, we further investigated the expression of *kiaa0319* in the notochord with fluorescence LSM combined with RNAscope probes targeting *kiaa0319* (Figure 2c). Longitudinal images at different developmental stages allowed us to accurately distinguish the spinal cord from the notochord (See also Video SV1 for the animation of a 3D reconstruction at 72 hpf and Figure S3 for the positive and negative controls). We detected much higher *kiaa0319* signal intensity in the notochord which became weaker as development progressed. Although weaker, a signal in the spinal cord was also observed. This was strongest at 96 hpf but, rather than increasing or stabilizing as development progressed, it became weaker at 120 hpf. Finally, analysis in the double Tg(gfap:GFP);Tg(Oligo2:dsRed) transgenic embryos further confirmed localization of *kiaa0319* expression in the notochord (Figure 2d). This transgenic line presents (a) secondary motor neurons, interneurons, and oligodendroglia cells labeled with GFP and (b) motor neurons and oligodendrocytes labeled with DsRed (Shin, Park, Topczewska, Mawdsley, & Appel, 2003) and therefore is useful to distinguish the developing neural tube.

**Figure 3:**
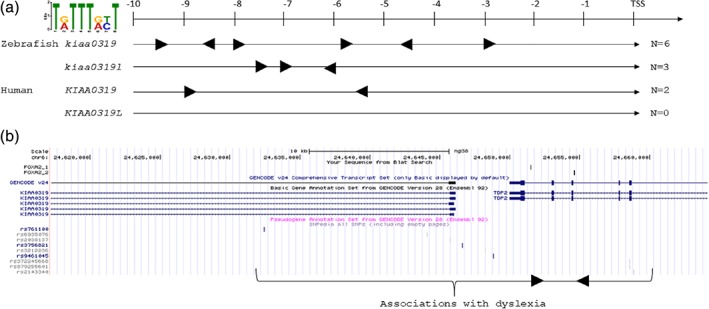
*FOXA2* consensus sequences at the *KIAA0319* and *KIAA0319‐LIKE* regulatory regions. (a) The 10 kb regions upstream of the transaction start sites (TSS) of *KIAA0319* and *KIAA0319L* in the zebrafish and human genomes were scanned for the presence of *FOXA2* consensus sequences (indicated in the top left corner). The results are visualized as black triangles with right and left orientation in reference to the positive and negative strand where the consensus sequence was found. The number of motifs is indicated at the right side of the figure. The exact position of the consensus sequences is shown in Table S3. (b) A snapshot from the UCSC genome browser shows the genomic location of the human *FOXA2* consensus sequences (top track). The two sequences are located in introns of *TDP2*, within the *KIAA0319* dyslexia‐associated region, indicated by the SNPs in the bottom track. The brace at the bottom provides a visualization of where the *FOXA2* sequences map within the dyslexia associations [Color figure can be viewed at wileyonlinelibrary.com]

Among the elements controlling gene expression in the notochord, *FOXA2* is a key transcription factor (Tamplin et al., 2011). We scanned the genomic sequences upstream of the *KIAA0319* and *KIAA0319L* TSS in both humans and zebrafish for *FOXA2* consensus motifs (Figure 3; Table S3). We analyzed the 10 kb region upstream of the TSS, where regulatory elements for downstream genes would most likely reside (Metzakopian et al., 2012). In zebrafish, six and three *FOXA2* motifs were found upstream of *kiaa0319* and *kiaa0319‐like,* respectively. Three of the six motifs upstream of *kiaa0319* were within the 6 kb upstream of the TSS, while all three of the FOXA2 motifs were more distant to the *kiaa319‐like* TSS. In humans, FOXA2 motifs were found only upstream of *KIAA0319* (*N* = 2) and not of *KIAA0319L*. Figure 3b shows the position of these two motifs relatively to *KIAA0319* and to the dyslexia‐associated SNPs (Newbury et al., 2014; Paracchini et al., 2006).

**Figure 4:**
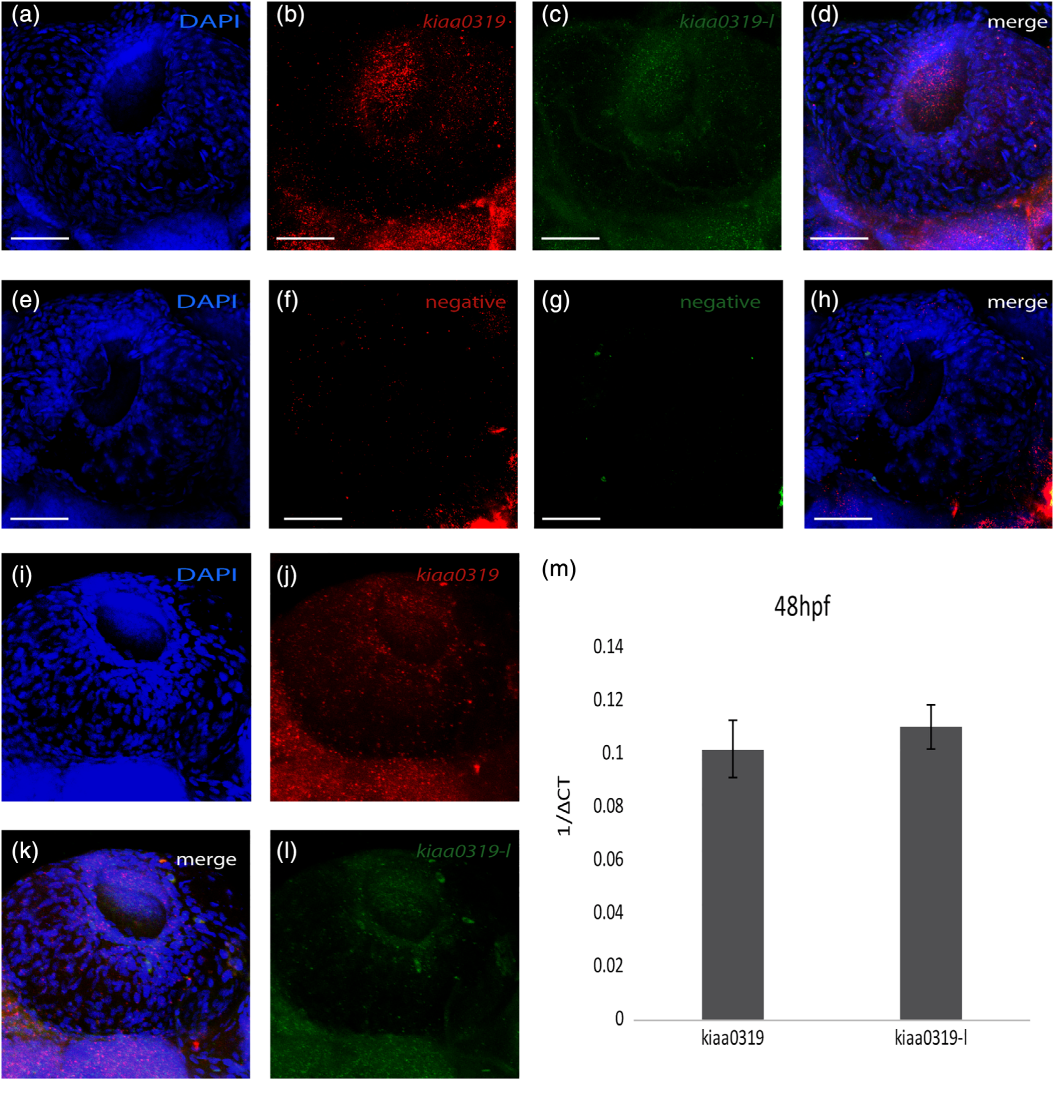
*kiaa0319* and *kiaa0319‐like* are expressed in the eyes. Results for RNAscope analysis in the eyes for *kiaa0319* (labeled in red; panels [b,j]) and *kiaa0319*‐*like* (labeled in green; [c,l]). The triple negative control shows no signal ([f,g] DAPI [a,e,i]) shows nuclear staining. Panels (d,h,k) show the merged signal for all channels. Panel (i) to (l) provide a different view of the eye. All images show the left side of animals oriented with brain on the left and tail on the right at 48 hpf (WT zebrafish). The scale bar is 50 μm in all panels. (m) Quantification of expression by qPCR of *kiaa0319* and *kiaa0319‐like*. Expression is measured as 1/ΔCt referenced against the *eef1a1l2* gene. The measurement is derived after pooling a total of 40 eyes collected at 48 hpf. The mean values were derived from three technical replicates. The error bars indicate standard deviations

Both the WISH and RNAscope analyses suggested expression of *kiaa0319* in the otic vesicles (Figure S4), which is of interest in the context of previous reports of a possible role of *Kiaa0319* in the auditory system of rodents (Centanni, Booker, et al., 2014; Centanni, Chen, et al., 2014; Guidi et al., 2017; Szalkowski et al., 2012). However, this structure tends to accumulate nonunspecific signal when conducting in situ hybridization because of technical artifacts, such as probe trapping. Detailed RNAscope analysis showed a signal for both *kiaa0319* (Figure 4b) and *kiaa0319‐like* (Figure 4c) in the otic vesicles, however a signal was detected also in the negative controls (Figure 4f,g). In comparison to the controls, both genes showed stronger expression and a signal characterized by a speckled pattern including within the main structures, suggestive of a genuine expression. In contrast, the controls showed a weaker signal, mainly localized along the contour of the otic vesicles suggesting probe trapping. However, given this background noise, we cannot conclude with confidence that *kiaa0319* and *kiaa0319l* are expressed at these structures.

The WISH analysis also suggested expression in the eyes, another structure that might lead to unspecific signals (Figure S2). The RNAscope analysis at the eyes showed expression for both *kiaa0319* (Figure 4b,j) and *kiaa0319‐like* (Figure 4c,l). Both genes are expressed on the surface of the eyes and, most strongly, around the eye lens. The negative controls had no signal confirming the specificity of the probes. Expression in the eyes was further confirmed by qPCR (Figure 4m).

## 4 DISCUSSION

We conducted the first zebrafish characterization of the dyslexia susceptibility *KIAA0319* gene. We found that *kiaa0319* is highly expressed at early developmental stages and, in addition to the expected expression in the brain; we show that it is expressed in the notochord, the eyes, and possibly the otic vesicles. For comparison, we analyzed the *kiaa0319‐like* gene, which showed a similar expression pattern but presented a very weak signal in the notochord. This observation is surprising given the generally higher and ubiquitous expression of *KIAA0319‐LIKE* reported in human tissues (Figure S1) and suggests a specific role for kiaa0319. To the best of our knowledge, this is the first study reporting expression of *kiaa0319* during the very first hours of development and clearly showing its expression in specific structures other than the brain.

The function of the KIAA0319 protein has been studied in human cell lines and in rodent models, however it is not yet fully understood. The first functional characterization was conducted in rats and suggested a role in neuronal migration (Paracchini et al., 2006) while more recent studies in mice indicate an involvement in biological processes beyond brain development (Franquinho et al., 2017; Guidi et al., 2017, 2018).

Consistent with the latest studies, we observed expression in the brain, but also observed expression in other organs. Guidi and colleagues (Guidi et al., 2017) generated a double KO mouse model for the *Kiaa0319* and *Kiaa0319l* genes and the most notable phenotype reported was an impairment of the auditory system. Analysis of individual KO for both genes showed mild effects for *Kiaa0319l* but no effects for *Kiaa0319* alone. Rodent models for other dyslexia candidate genes (i.e., *Dcdc2* and *Dyx1c1*) have also suggested an impairment in auditory processing (Szalkowski et al., 2013; Truong et al., 2014). The potential expression of both *kiaa0319* and *kiaa0319l* in the otic vesicles (Figure 2, Figures S2 and S4) would be interesting in the context of the rodent data. However, further work will be required to establish whether these genes are expressed at the otic vesicles given possible probe trapping in these structures (Figure S4). Instead, given the eye‐specific expression observed in our study (Figure 4), it would be valuable to assess visual phenotypes in rodent models.

Whether dyslexia is the result of a deficit in sensory systems, as predicted by the magnocellular theory (Stein, 2001), remains highly debated (Paracchini et al., 2016). Defects in both the visual and auditory systems have been reported in individuals with dyslexia across different studies, but heterogeneity and inconsistency across studies remain significant challenges (Goswami, 2014). The *kiaa0319* expression in the eyes during zebrafish development could be considered in line with a role in sensory organs. While it would be tempting to reach conclusions, it is worth noting that it is not possible to generalize and make strong assumptions based on observations for genes analyzed in isolation. Moreover, the *KIAA0319* genetic associations (as with most genetic associations and with complex traits) explains only a small fraction of dyslexia heritability (Paracchini et al., 2016).

The most compelling observation of our study is the strong notochord expression of *kiaa0319*, which, in comparison, was only very weak for *kiaa0319‐like* (Figure 2). The notochord is a transient embryonic structure in zebrafish essential for guiding the development and patterning of the early embryo (Stemple, 2005). Because of the important functions of the notochord, the identification of the notochord‐expressed genes is important to understand these developmental processes. The notochord is a source of signaling to the surrounding tissues to guide structural development, particularly for the spinal cord. For example, the notochord is the source of sonic hedgehog (SHH) signaling which controls many processes including the development of motor neurons, the establishment of the dorsal‐ventral axis and left/right asymmetries (Echelard et al., 1993; Schilling, Concordet, & Ingham, 1999). The notochord also controls, in a highly specific spatiotemporal manner, the trajectories of dorsal root ganglion (DRG) axons through repressive signals mediated by aggrecan, one of the chondroitin sulfate proteoglycans (CSPGs) specifically found in the cartilage (Masuda et al., 2004). A similar repressive role has been described for *Kiaa0319* in mice, including the repression of axon growth in hippocampal and DRG neurons (Franquinho et al., 2017). The same study also showed that *Kiaa0319* was expressed in sensory and spinal cord neurons in postnatal and adult mice. Our data are consistent with these findings suggesting an evolutionary conserved function for *kiaa0319* beyond brain development.

While it is not possible to directly infer information about gene function from expression patterns, our data represent a useful resource to guide and interpret follow up functional studies. First of all, the specific spatial/temporal pattern suggests that zebrafish would be a valuable model for knock‐down and knock‐out studies. Furthermore, these results will help to formulate well‐defined hypotheses on which assays and which lines or mutants might be particular useful at dissecting specific roles at well‐defined structures.

A specific expression pattern is likely to result from a fine‐tuned regulation. Most of the markers associated with dyslexia map to the *KIAA0319* regulatory regions (Figure 3; Newbury et al., 2014; Paracchini et al., 2006). Previously, we showed that a dyslexia‐associated allele (rs9406145) at this region affects the affinity for a transcription factor and reduce the expression of *KIAA0319* (Dennis et al., 2009). *FOXA2* is a key transcription factor involved in the regulation of genes expressed in the notochord (Tamplin et al., 2011). However, *FOXA2* motifs are not sufficient to predict expression in the notochord as other transcription factors (e.g., Brachyury, GLIS3, and RFX3) might be required and would function through enhancers located at specific distances (Farley, Olson, Zhang, Rokhsar, & Levine, 2016). Nevertheless, the FOXA2 consensus motifs upstream of *KIAA0319* TSS support the patterns observed in Figure 2 showing a much stronger expression for *kiaa0319* in the notochord compared to *kiaa0319l*. The region upstream of *kiaa0319* had more FOXA2 consensus sequences and in more proximal position compared to *kiaa0319l*. In humans, FOXA2 motifs were found for *KIAA0319* only, suggesting a conserved role for *KIAA0319* across vertebrates. These observations also provide a framework to interpret the genetic associations with dyslexia reported in *KIAA0319* noncoding regions (Figure 3) (Dennis et al., 2009; Paracchini et al., 2006). Genetic variation at this locus might affect not only gene expression levels, as previously shown (Dennis et al., 2009), but also perturb the regulation of a specific spatiotemporal pattern.

In summary, our characterization of the *KIAA0319* dyslexia susceptibility gene in zebrafish reveals a specific pattern of expression during development. In addition to the expected expression in the brain, we show for the first time high embryonic expression during the first hours of development and, later on, at specific structures such as the eyes and the notochord. Our study therefore supports a developmental role for *KIAA0319* which is not restricted to the brain and may contribute to the ongoing discussion around the role of neuronal migration in dyslexia. While our data do not exclude a role in the developing brain and in neuronal migration, they suggest an involvement in other developmental processes as well.

## CONFLICT OF INTEREST

The authors declare no competing interests.

## DATA AVAILABILITY STATEMENT

The data that support the findings of this study are presented in the main text and supplementary material. Additional data and images are available from the corresponding author.

## Supporting information


**Appendix S1**: Supplementary Methods

**Supplementary Video V1 Animation of a 3D reconstruction showing kiaa0319 expression in the notochord.** The animation has been reconstructed from light‐sheet microscopy images collected at 72 hpf as shown in Figure 2 [c] in main text. The video indicate to position of the notochord relative to the spinal chord. The green signal is given by a RNAscope probe specific to *kiaa0319*. For the positive and negative control see Supplementary Figure S3
