# Supplementary material for: The dyslexia susceptibility KIAA0319 gene shows a specific expression pattern during zebrafish development supporting a role beyond neuronal migration
Source: J Comp Neurol. 2019 Apr 16;527(16):2634–43. doi: 10.1002/cne.24696 (PMC6767054; doi:10.1002/cne.24696)
Supplement: Supplementary file 1 — Appendix S1: Supplementary Methods [file CNE-527-2634-s001.docx]

**The dyslexia susceptibility KIAA0319 gene shows a highly specific expression pattern during zebrafish development supporting a role beyond neuronal migration.**

**Supplementary Methods`**

*Quantitative PCR.* Gene expression was assessed by quantitative PCR (qPCR) conducted with the Luna Universal RT-qPCR Kit (NEB) and using a Viia7 instrument (Life Technologies, Paisley, UK). Cycling conditions were as follows: 95°C for 1’ followed by 40 cycles of denaturing (95°C) for 10’’ and annealing for 30’’ (60°C). The primer pairs were designed to span across *kiaa0319* and *kiaa0319-like* exons. Reaction efficiency was calculated using a 5-point standard curve generated by 2 fold dilutions starting from 50 ng. A no-template sample was included as negative control. Melt curve analysis showed a single clear peak for each assay. Gene expression was plotted as 1/∆Ct referenced against the *eef1a1l2 gene* with each gene expression value derived from at least three technical replicates. All primers used are listed in Supplementary Table S1 and were designed from the annotations of the University of California, Santa Cruz (UCSC) and ENSEMBL genome browsers.

*Light-sheet microscopy*. We used an in-house built light-sheet microscope based on the OpenSPIM design (2). The original setup was modified to achieve an inverted configuration to accommodate larger varieties of samples. The microscope fits on a 450 mm × 300 mm breadboard (MB3045/M, Thorlabs), as in the original OpenSPIM design. A 488 nm wavelength laser (Solstis with frequency doubler, M Squared) provides the illumination through a single mode fibre. A beam expander is followed by an adjustable slit (VA100/M, Thorlabs) to control the width of the beam and a cylindrical lens (LJ1695RM-A, FL 50mm, Thorlabs) to focus the beam into a sheet of light. A steering mirror directs the beam to the illumination objective (UMPLFLN 10XW, water dipping, NA=0.3, Olympus) through a relay lens. The two objectives are mounted on a customized holder which not only simplifies the system but also minimizes adjustment required. This holder also allows a change of objective lens if needed. The fluorescence signal is first collected by a detection objective (LUMPLFLN 20XW NA=0.5, water dipping, Olympus) coupled with an achromatic lens (as tube lens, LA1708-A-ML, FL 200 mm, Thorlabs) and is then projected onto a sCMOS camera (ORCA-Flash4.0 sCMOS camera, Hamamatsu).

The imaging was performed by scanning the sample through the light sheet with a manual scanning stage. The 3D images were reconstructed by choosing an arbitrary z step using ImageJ (3) or through the VTK library Visualization Tool Kit (4) in the open source software Icy (5).

**Supplementary Table S1. List of primers**

| **Gene** | **Zebrafish gene name** | **ENSEMBL gene ID** | **Sequences** | **Fragment size (bp)** | **Experiment** |
| --- | --- | --- | --- | --- | --- |
| *kiaa0319* | si:ch73-215d9.1 | ENSDARG00000103001 | AGGGTCAGTACACGTTTCAGC | 1024 | RT-PCR |
|  |  |  | CACAGAGGGTCACAGGAACAG |  |  |
| *kiaa0319* |  |  | AACCATCGCTGTGAAAAGGC | 121 | qPCR |
|  |  |  | CTTTCAGAGTAGGTTGCGGC |  |  |
| *kiaa0319* |  |  | AGGGTCAGTACACGTTTCAGC | 1066 | WISH probe |
|  |  |  | CGCAATTAACCCTCACTAAAGGGACACAGAGGGTCACAGGAACAG |  |  |
| *kiaa0319-like* | si:ch211-193k19.1 | ENSDARG00000035660 | CGCAGCCACATGTAGAGTCT | 119 | qPCR |
|  |  |  | AGAAGACATGTCCTGCTCCG |  |  |
| *β-actin2* | actb2 | ENSDARG00000037870 | GCAGAAGGAGATCACATCCCTGGC | 322  (407, genomic) | RT-PCR |
|  |  |  | CATTGCCGTCACCTTCACCGTTC |  |  |
| *eef1a1l2* | eef1a1l2 | ENSDARG00000020850 | TTGAGAAGAAAATCGGTGGTGCTG | 91 | qPCR |
|  |  |  | GGAACGGTGTGATTGAGGGAAATTC |  |  |

**Supplementary Table S2. PFA treatment duration for the RNAscope protocol**

| **Stage** | **Pre-treatment (min)** | **PFA treatment (h)** |
| --- | --- | --- |
| >12hpf |  | 4 (with chorion) |
| 12hpf | 1.5 | 1 (with chorion) |
| 24hpf | 3 | 0.5 (without chorion) |
| 36hpf | 5 | 0.5 |
| 48hpf | 7 | 0.5 |
| 72hpf | 10 | 0.5 |
| 96hpf | 12 | 0.5 |
| 120hpf | 15 | 0.5 |

**Supplementary Table S3. Location of FOXA2 consensus sequences**

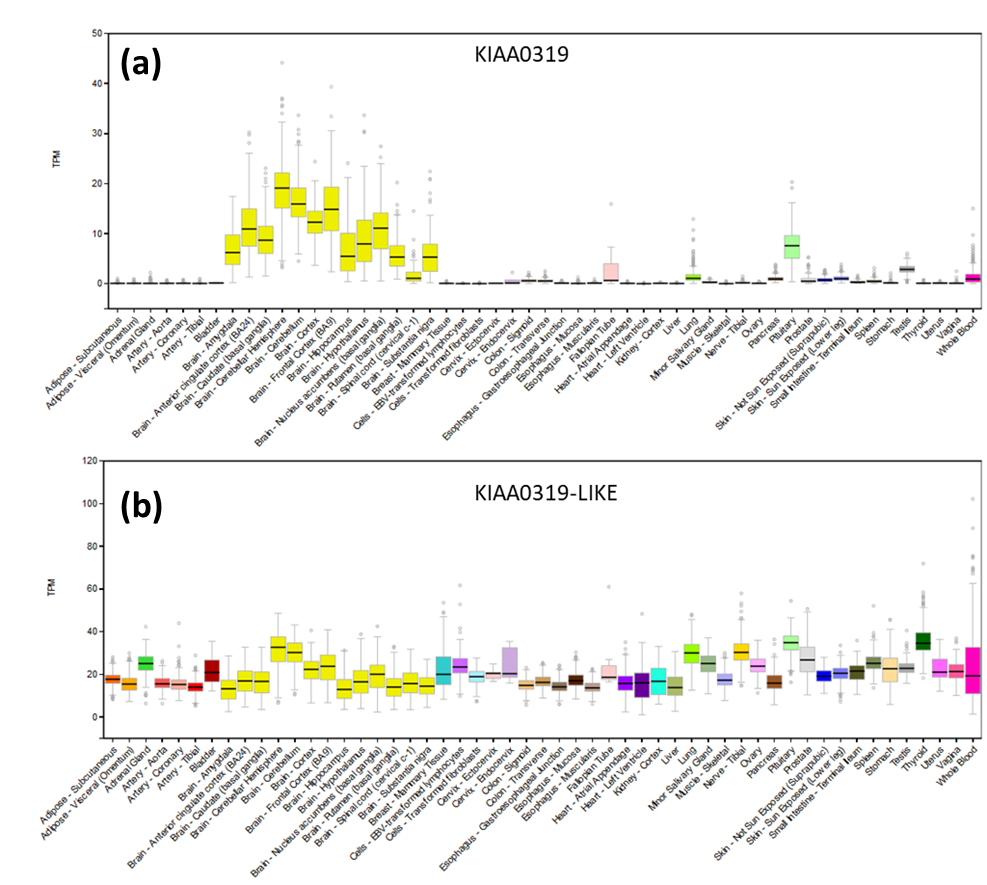


**Supplementary Figure S1. *KIAA0319* is specifically expressed in the human brain.** Expression profiles across human adult tissues are shown for the *KIAA0319* (A) and the *KIAA0319-LIKE* (B) genes. *KIAA0319* is specifically expressed in the adult brain. In comparison, *KIAA0319-LIKE* expression is higher, including in the brain, and widespread across tissues. The images are screenshots following queries to the GTEx database using the default settings (6). TPM = transcript per million.

**
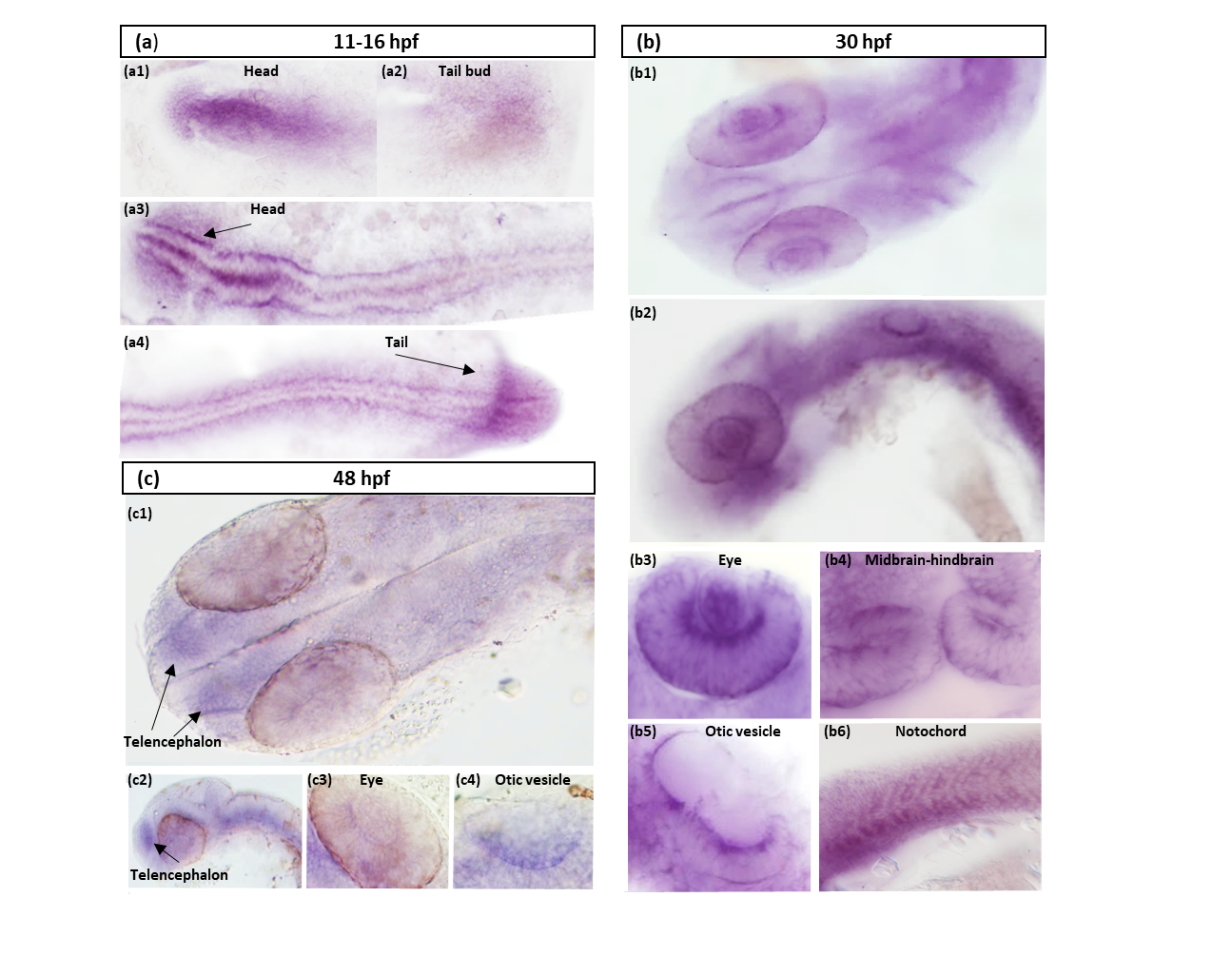
**

**Supplementary Figure S2.** ***In situ* hybridization suggests a specific spatiotemporal expression pattern for *kiaa0319*. (a)** Expression up to 16 hpf. At the 3 somite stage, *kiaa0319* is expressed throughout the embryo with the strongest signal in the head **(a1)** and to a lesser extent, in the tail bud **(a2)**. At the 14 the somite stage, high expression continues in the head **(a3)** and is visible along the developing body midline and in the tail **(a3) (a4)**. All images are dorsal views with the head on the left side. At 30 hpf **(b)** *kiaa0319* is still expressed throughout the embryo but strong expression emerges in specific structures observed from dorsal **(b1)** and lateral **(b2)** views. Details of expression are shown for the eyes (**b3;** dorsolateral view), the midbrain-hindbrain boundary (**b4;** dorsal view), the otic vesicles (**b5**; dorsolateral view) and the notochord (**b6**; lateral view). In a dorsal view at 48 hpf **(c)**, *kiaa0319* expression in the eyes is diminished, while signal in the telencephalon emerges **(c1)**. The signal in the telencephalon is particularly visible in a lateral view **(c2)** along with expression in the eyes and the region around the notochord. Detailed dorsolateral views of the eye **(c3)** and otic vesicle **(c4)** show weaker intensity at these structures when compared to the pattern observed at 30 hpf.

**
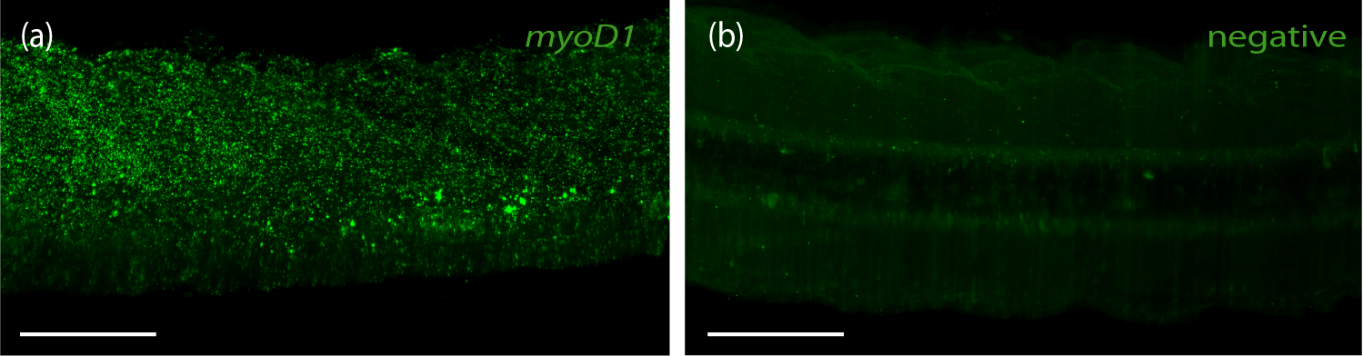
**

**Supplementary Figure S3. Controls for the RNAScope Fluorescent Multiplex Assay imaging by light sheet microscopy.** For all experiments a probe for *myoD1* (myogenic differentiation 1) was used as a positive control **(a)** and three unspecific probes for each of the three channels were used as a triple negative control **(b)**. The positive control gives the characteristics dotted signal while the signal detected in the negative panel is simply due to auto-florescence. The images are a reference for the results presented in Figure 2(c) picture showing longitudinal view of the body at 120 hpf taken with the settings used for the detection of the *kiaa0319*probe. The scale bar indicates 50 µm in all panels.


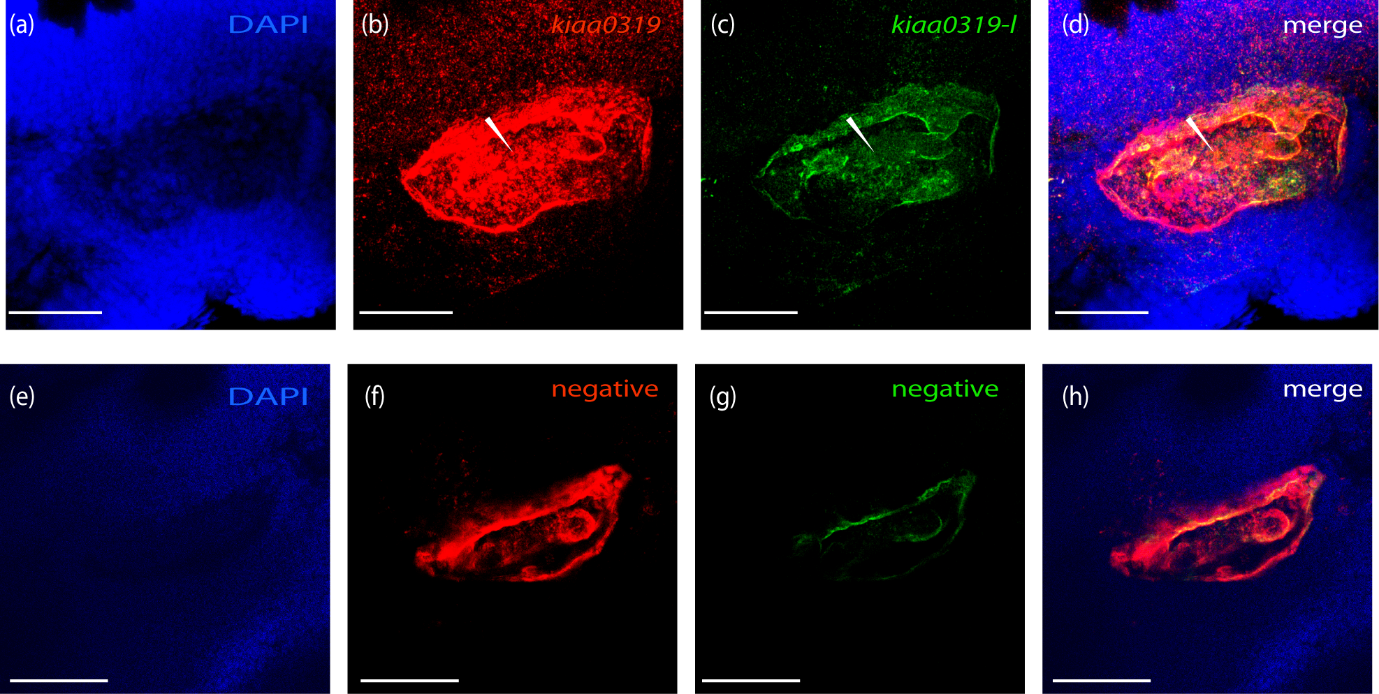


**Supplementary Figure S4**.  ***RNAscope analysis at the otic vesicles.*** *kiaa0319,* labelled in red **(b)**, and *kiaa0319*-*like*, labelled in green **(c)** are compared against the negative controls **(f)(g)**. The signal for both *kiaa0319* and *kiaa0319-like* is characterised by the presence of speckles (white arrows; **(b)(c)**). The signal around the otic vesicles (especially for *kiaa0319* in the top part of the **(b)** panel) show expression in the brain confirming the specificity of the probes. In contrast, in the triple negative control **(f)(g)**, most of the signal is confined around the otic vesicles structures and could be due to probe trapping at the contour. DAPI **(a)(e)** shows nuclear staining and the merged signal for all channels is shown in **(d)(h)**. All images show the left side of WT zebrafish at 48 hpf oriented with brain on the left and tail on the right. The scale bar is 50 µm in all panels.

**Supplementary Video V1. Animation of a 3D reconstruction showing kiaa0319 expression in the notochord.** The animation has been reconstructed from light-sheet microscopy images collected at 72 hpf as shown in fig 2 (c) in main text. The video indicate to position of the notochord relative to the spinal chord. The green signal is given by a RNAscope probe specific to *kiaa0319*. For the positive and negative control see Supplementary Figure S3

**References**

1. Schmittgen,T.D. and Livak,K.J. (2008) Analyzing real-time PCR data by the comparative CT method. *Nat. Protoc.*, **3**, 1101–1108.

2. Pitrone,P.G., Schindelin,J., Stuyvenberg,L., Preibisch,S., Weber,M., Eliceiri,K.W., Huisken,J. and Tomancak,P. (2013) OpenSPIM: an open-access light-sheet microscopy platform. *Nat. Methods*, **10**, 598–599.

3. Schindelin,J., Arganda-Carreras,I., Frise,E., Kaynig,V., Longair,M., Pietzsch,T., Preibisch,S., Rueden,C., Saalfeld,S., Schmid,B., *et al.* (2012) Fiji: an open-source platform for biological-image analysis. *Nat. Methods*, **9**, 676–682.

4. Schroeder,W., Martin,K., Lorensen,B. and Kitware,I. (2006) The visualization toolkit : an object-oriented approach to 3D graphics Kitware.

5. de Chaumont,F., Dallongeville,S., Chenouard,N., Hervé,N., Pop,S., Provoost,T., Meas-Yedid,V., Pankajakshan,P., Lecomte,T., Le Montagner,Y., *et al.* (2012) Icy: an open bioimage informatics platform for extended reproducible research. *Nat. Methods*, **9**, 690–696.

6. Consortium,G.Te. (2013) The Genotype-Tissue Expression (GTEx) project. *Nat Genet*, **45**, 580–585.
